# Monoclonal antibody therapy for Kawasaki disease: a protocol for systematic reviews and meta-analysis

**DOI:** 10.1186/s13643-016-0236-2

**Published:** 2016-04-12

**Authors:** Osamu Nomura, Sayaka Fukuda, Erika Ota, Hiroshi Ono, Akira Ishiguro, Tohru Kobayashi

**Affiliations:** Division of Pediatric Emergency Medicine, Tokyo Metropolitan Children’s Hospital, 2-8-29, Musasidai, Fuchu-shi, Tokyo 183-8561 Japan; Department of Pediatrics, Kawakita General Hospital, 1-7-3, Asagaya-kita, Suginami-ku, Tokyo 166-0001 Japan; Department of Health Policy, National Center for Child Health and Development, 2-10-1, Okura, Setagaya-ku, Tokyo 157-8535 Japan; Division of Cardiology, National Center for Child Health and Development, 2-10-1, Okura, Setagaya-ku, Tokyo 157-8535 Japan; Department of General Pediatrics and Interdisciplinary Medicine, National Center for Child Health and Development, 2-10-1, Okura, Setagaya-ku, Tokyo 157-8535 Japan; Division of Clinical Research Planning, Department of Development Strategy, Center for Clinical Research and Development, National Center for Child Health and Development, 2-10-1, Okura, Setagaya-ku, Tokyo 157-8535 Japan

**Keywords:** Kawasaki disease, Monoclonal antibody agents, Coronary artery abnormality, Meta-analysis, Systematic review

## Abstract

**Background:**

Kawasaki disease (KD) is a form of self-limiting vasculitis that causes coronary artery abnormality in children. Based on reports of elevated plasma level of cytokines such as tumor necrosis factor-α in KD patients, clinical trials of monoclonal antibodies that block cytokine cascades have been conducted. However, the studies have revealed contradictory results. The objective of this study is to examine the effectiveness of treatment with monoclonal antibodies for KD patients.

**Methods:**

Relevant randomized controlled trials (RCTs), cluster RCTs, quasi-RCTs, cross-over trials, and any observational studies (e.g., cohort studies, case-control studies, case series, and case reports) will be included to summarize available evidence both qualitatively and quantitatively. Cochrane Central Register of Controlled Trials, MEDLINE, EMBASE, and ICUSHI will be searched. We will assess coronary artery and treatment outcomes of the interventions. Two authors will independently screen studies for inclusion and consulting with a third author where necessary to resolve discrepancies. The risk of bias of included studies will be assessed using the Cochrane Collaboration risk of bias tool and quality of evidence using the Grading of Recommendations Assessment, Development and Evaluation (GRADE) approach. Meta-analysis of the included studies will be conducted using fixed effects or random effects models depending on the degree of between-study heterogeneity. Results will be presented using risk ratios with 95 % confidence interval (CI) for dichotomous outcomes and standardized mean differences with 95 % CI for continuous outcomes.

**Discussion:**

This systematic review and meta-analysis protocol does not require ethical approval. We will disseminate the findings of this systematic review and meta-analysis via publications in peer-reviewed journals.

**Trial registration:**

PROSPERO CRD42016033079.

**Electronic supplementary material:**

The online version of this article (doi:10.1186/s13643-016-0236-2) contains supplementary material, which is available to authorized users.

## Introduction

Kawasaki disease (KD) is a self-limiting form of vasculitis of unknown cause and is a major cause of acquired heart disease in developed countries [1]. KD was originally described in 1967 by Tomisaku Kawasaki and mainly affects children less than 5 years of age, with peak onset between 10 and 12 months [2, 3]. The incidence of KD in children younger than 5 years old varies; 239.6 per 100,000 in Japan, 17.1 per 100,000 in the USA, and 8.1 per 100,000 in the UK [4]. Diagnosis of KD is generally based on the clinical signs and symptoms described in either of the two major sets of criteria. The diagnostic guidelines of the Japan KD Research Committee require any five signs or symptoms including (1) fever longer than 5 days, (2) bulbar nonexudative conjunctival injection [5] and cervical lymphadenopathy, (4) polymorphous rash, (5) oral and perioral changes, and (6) extremity changes [6].

Although the cause of KD is still unknown, the main pathophysiology has been found to be associated with systemic vasculitis, particularly affecting coronary arteries, which leads to the development of coronary artery aneurysms (CAAs) in 15–25 % of untreated patients. About 2–3 % of untreated KD cases die as a result of coronary vasculitis [3]. Therefore, there has been intense interest in treatments to reduce the risk of CAAs. KD is also potentially an important cause of long-term cardiac diseases in adult life. Treatment of acute stage KD with aspirin and intravenous immunoglobulin (IVIG) has been established as a standard initial therapy, which has been shown to limit the duration of the acute phase of KD, as well as reducing the incidence of long-term coronary event from 25 % to less than 3 % [7, 8]. However, about 20 % of patients had persistent or recurrent fever after completion of IVIG, together with a particularly high risk of developing CAAs [9]. Therefore, intensification of the initial therapy and second-line therapy has been the focus of clinical research for acute phase treatment of KD.

Currently, there are reports on the elevation of plasma levels of a variety of multiple inflammatory cytokines, such as tumor necrosis factor-α (TNF-α), interleukin (IL)-1b, IL-6, and IL-8 during the acute phase of KD [10]. Among these cytokines, TNF-α was found to play an important role in the development of coronary artery lesions in mouse models of KD [11]. Clinical trials of TNF-α-related biologics such as infliximab and etanercept as an initial or additional rescue therapy for refractory KD patients yielded contradictory results [12–14]. Moreover, studies on treatments with other monoclonal antibodies such as anakinra, tocilizumab, and rituximab are limited to case reports or case series [15–18]. Taken together, these studies suggest that the effectiveness of monoclonal antibody therapeutic agents for acute KD remain undetermined. To that end, we will conduct a systemic review and meta-analysis to summarize the evidence of the potential effectiveness and safety of monoclonal antibodies as a treatment option for KD patients.

## Objectives

The main objective of this systematic review is to evaluate the effectiveness of monoclonal antibodies as therapeutic agents for KD patients. We will investigate the effectiveness of initial or additional treatment with monoclonal antibodies for KD patients towards the incidence of CAAs or patients who need additional rescue therapy, compared with conventional aspirin with IVIG treatment.

## Methods/design

### Eligibility criteria

#### Types of studies

Our systematic review and meta-analysis will include randomized control trials (RCTs) and cluster RCTs, quasi-RCTs, cross-over trials, and any observational studies (e.g., cohort studies, case-control studies, case series, and case reports).

This protocol is registered with PROSPERO (International prospective register of systematic reviews) at the National Institute for Health Research and Centre for Reviews and Dissemination (CRD), University of York (registration number: CRD42016033079).

#### Types of participants

We will include all patients diagnosed with KD globally in the analysis. The diagnosis of KD includes both complete and incomplete KD and must fulfill the diagnostic guidelines of the Japan KD Research Committee or the American Heart Association guidelines [7, 19].

#### Types of intervention

All forms of monoclonal antibody therapies in conjunction with any combination of placebo or no treatment, IVIG, aspirin, or corticosteroid for the treatment of KD will be considered as the intervention of interest.

#### Types of outcome measures

##### Primary outcomes

1. Coronary artery abnormalities.
  The incidence of aneurysmal coronary abnormalities (measured via diameter or *z*-scores) per study group found at either coronary angiogram or echocardiography within 3 months of KD diagnosis. The definition of coronary abnormality was defined by either the Z-score (a coronary dimension that is ≥2.5 standard deviations (SDs) above the mean for that body surface area) or the Japanese Ministry of Health criteria [20]:
  - Lumen >3 mm in children <5 years old;
  - Lumen >4 mm in children >5 years old;
  - Internal diameter of a segment measuring ≥1.5 times that of an adjacent segment.
2. Treatment resistance.
  Treatment resistance is defined as a clinical state of non-responding to an initial therapy.

##### Secondary outcomes

1. Duration of fever (days)
2. Length of hospital stay (days)
3. Time for biochemical parameters to normalize: CRP and ESR
4. Incidence of adverse event (for example, fever, coronary artery abnormalities, headache, and hemolytic anemia)
5. The incidence of any adverse event per study group that is attributable to the administration of monoclonal antibodies at any point after treatment initiation

#### Electronic searches

We will conduct the systematic review and meta-analysis according to the Cochrane Handbook for Systematic Reviews of Interventions and the Meta-analysis of Observational Studies in Epidemiology (MOOSE) census statement. This protocol follows the Preferred Reporting Items for Systematic Review and Meta-Analysis Protocols 2015 (Additional file 1) [21].

A comprehensive literature review using the database of the Ovid platform including MEDLINE, EMBASE plus EMBASE classics and Cochrane Central Register of Controlled Trials, and ICHUSHI (research database in Japanese conducted by the Japan Medical Abstracts Society) will be performed. Literature search strategies will be developed using medical subject headings (MeSH) and text related to “Kawasaki disease” and “monoclonal antibody.” We will include all languages in our searches. Our search strategy will be reviewed by an experienced librarian at the National Center for Child Health and Development. Additional file 2 shows the search strategy in more detail.

#### Searching other resources

The International Clinical Trials Registry Platform Search Portal, ClinicalTrials.gov, and UMIN-CTR will be searched for ongoing or recently completed trials.

#### Data collection and analysis

- Eligible criterion
  1. Studies which examined the efficacy and/or safety of monoclonal antibodies (e.g., infliximab, etanercept, rituximab, anakinra, and tocilizumab) for patients with KD.
- Ineligible criteria
  1. Experimental studies (in vivo study)
  2. Studies with no relationship with Kawasaki disease
  3. Studies with no relationship with monoclonal antibodies
  4. Unoriginal studies (e.g., review, news, editorial, comment)
  5. Studies with no outcomes of interest
  6. Duplicated studies

#### Data extraction and management

Two reviewers (ON and SF) will independently screen titles and abstracts of all the retrieved bibliographic records. The eligible and ineligible criteria will be used for each screening step. If no abstract is available, the full text will be obtained unless the article can be confidently excluded by its title alone. If there is any doubt whether a study should be excluded, the study will proceed to the full-text screen to reduce the likelihood of incorrectly excluding relevant studies. Full text of potentially eligible studies will be independently retrieved by the two reviewers. Disagreements at these screening levels (title/abstract and full text) will be resolved through discussion with or adjudication by a third reviewer (TK).

#### Data collection

A standard set of data will be extracted for each study using a tailored data extraction form.

1. General study identification.
  First author, year of publication, publication type, and country where the study took place.
2. Fulfillment of eligibility criteria.
  Study type, interventions, outcomes measured, and reasons for exclusion.
3. Participants.
  Number of enrolled patients, age, sex, race, KD severity, and eligibility criteria.
4. Intervention (monoclonal antibodies).
  Type of monocle antibody, dosing, frequency, timing (as initial therapy or additional rescue therapy), and concomitant treatment.
5. Outcomes.
  Coronary diameters, coronary abnormality, treatment resistance, adverse events, duration of fever, duration of laboratory parameter abnormality (e.g., CRP and/or ESR), and length of hospital stay.

#### Assessment of risk of bias in included studies

The risk of bias in included studies will be assessed using the Cochrane risk of bias tool according to the handbook and the Risk of Bias Assessment Tool for Non-Randomized Studies (RoBANS) for observational studies [22, 23]. We will use the following criteria to assess the risk of bias: random sequence generation, allocation sequence concealment, blinding of participants and personnel, blinding of outcome assessment, incomplete outcome data, selective outcome reporting, and other biases. In addition, we will assess the method as high risk of bias, low risk of bias, and unclear risk of bias.

#### Measurement of treatment effect

Studies will be included in meta-analysis if they are of the same type, such as RCTs or cluster RCTs, and have the same population, intervention, comparison, and outcomes. Evaluation of whether included studies are eligible for meta-analysis will be conducted by two authors (ON and SF), and in the event of disagreement, the disagreement will be resolved through discussion with or adjudication by a third reviewer (TK).

We will narratively summarize the available evidence when it is not possible to perform a meta-analysis.

1. Dichotomous data.
  We will present results as summary risk ratio with 95 % confidence interval (CI) in the analysis of RCTs and present results as odds ratio with 95 % CI in the analysis of observational studies.
2. Continuous data.
  We will use the mean difference with 95 % CIs if outcomes were measured in the same way between trials. We will use the standardized mean difference with 95 % CIs to combine trials that measured the same outcome but used different methods.

#### Unit of analysis issues

1. Cluster-randomized trials.
  We will include cluster-randomized trials in the analyses along with individually randomized trials. We will adjust their sample sizes using the methods described in the handbook and an estimate of the intracluster correlation coefficient (ICC) derived from the trial (if possible), from a similar trial or from a study of a similar population. If we use ICCs from other sources, we will report this and conduct sensitivity analyses to investigate the effect of variation in the ICC. If we identify both cluster-randomized trials and individually randomized trials, we plan to synthesize the relevant information. We will consider it reasonable to combine the results from both if there is little heterogeneity between the study designs, and the interaction between the effect of intervention and the choice of randomization unit is considered to be unlikely. We will also acknowledge heterogeneity in the randomization unit and perform a sensitivity analysis to investigate the effects of the randomization unit.
2. Multi-armed trials.
  Multi-armed trials will be included in the analyses. We will combine all relevant interventions into a single group and incorporate all relevant control groups into a single group. Any other different interventions will be addressed in different meta-analyses. If one of the arms is irrelevant, we will exclude it from the analysis.

#### Management of missing data

Levels of attrition will be recorded for the included studies. We will explore the impact of included studies with high levels of missing data in the primary outcome by using sensitivity analysis. For all outcomes, intention-to-treat (ITT) analysis will be used as much as possible. All participants will be analyzed in the group to which they were allocated, regardless of whether they received the allocated intervention.

#### Strategy for data synthesis

Statistical analysis will be performed using Review Manager V.5.3 (Cochrane Collaboration software). If the collected data from included studies show statistical homogeneity, fixed effects meta-analysis will be performed. If the data are anticipated to have significant heterogeneity between studies, we will perform random effects meta-analyses if an average treatment effect across trials is considered clinically meaningful. The results of the random effects model will be used as the average range of possible intervention effects with 95 % CIs and the estimates of *I*^*2*^, *T*^*2*^, and *χ*^*2*^, and the difference in clinical implication between interventions will be discussed. Finally, we will assess the quality of the following individual outcomes and produce summaries using the Grading of Recommendations Assessment, Development, and Evaluation (GRADE) approach:

1. Coronary artery abnormalities
2. Treatment resistance
3. Incidence of adverse event
4. Duration of fever
5. Length of hospital stay

Data will be imported from RevMan 2014 to the GRADE profiler to produce “summary of findings” tables. These tables will include a summary of the intervention effect and a quality of individual outcomes using the GRADE approach. The quality of the body of evidence for each outcome will be assessed based on five factors and will be downgraded from *high quality* by one level for serious or by two levels for very serious limitations: study limitations, consistency of effect, imprecision, indirectness, and publication bias.

#### Assessment of heterogeneity

We will evaluate heterogeneity in the meta-analyses using *I*^*2*^, *T*^*2*^, and *χ*^*2*^ statistics. We will consider that heterogeneity exists if *I*^*2*^ is 50 % or more, *T*^*2*^ is greater than 0, or when the significance of *χ*^*2*^ is lower than 0.10.

#### Assessment of reporting bias

If there are sufficient studies (10 or more) in the meta-analysis, we will investigate reporting biases (publication biases) using funnel plots. If asymmetry is identified or found in a visual assessment, the asymmetry will be verified using exploratory analyses.

#### Subgroup analysis and investigation of heterogeneity

We will implement subgroup analyses of the following:

1. Type of monoclonal antibody: infliximab vs. others
2. Country of origin: Japan vs. other countries
3. Timing of intervention: initial therapy vs. additional rescue therapy for IVIG non-responders

#### Sensitivity analysis

We will perform sensitivity analysis if the review might affect the results due to the high risk of bias of some of the included trials. For the purpose of this sensitivity analysis, we will define *high quality* as a trial having a low risk of random sequence generation, adequate allocation concealment, and the percentage of missing data less than 20 %, given the stated importance of attrition as a quality measure. Only the primary outcome will be included in the sensitivity analyses. If statistical heterogeneity exists in outcomes, we will carry out the sensitivity analysis to explore the effects of fixed or random effects analyses. Furthermore, if there are any assumptions for ICC values used in cluster-randomized trials, we will perform sensitivity analysis.

## Discussion

This review and meta-analysis will provide evidence of the effectiveness of monoclonal antibodies as a therapeutic option for KD patients. Additionally, our review will guide the future development of clinical and basic research in the management of KD.

## Additional files

Additional file 1: PRISMA-P checklist: recommended items to address in a systematic review protocol. (DOC 81 kb) Additional file 2: Search terms and strategies. The search strategy utilized is outlined in more detail in the file. (DOCX 41 kb)

## Abbreviations

CAA: coronary artery aneurysm
IL: interleukin
IVIG: intravenous immunoglobulin
KD: Kawasaki disease
TNF-α: tumor necrosis factor-α
