# Supplementary material for: Monoclonal antibody therapy for Kawasaki disease: a protocol for systematic reviews and meta-analysis
Source: Syst Rev. 2016 Apr 12;5:60. doi: 10.1186/s13643-016-0236-2 (PMC4830038; doi:10.1186/s13643-016-0236-2)
Supplement: Additional file 2: — Search terms and strategies. The search strategy utilized is outlined in more detail in the file. (DOCX 41 kb) [file 13643_2016_236_MOESM2_ESM.docx]

Additional file 2: Search terms and strategies

1) Medline

1 Mucocutaneous Lymph Node Syndrome/

2 mucocutaneous Lymph Node.tw.

3 kawasaki syndrome*.tw.

4 kawasaki disease*.tw.

5 or/1-4

6 exp Antibodies, Monoclonal/

7 ("monoclonal antibody" or "monoclonal antibodies").tw.

8 infliximab*.tw.

9 Remicade*.tw.

10 Inflectra*.tw.

11 Revellex*.tw.

12 Remsima*.tw.

13 Avakine*.tw.

14 TNF*.tw.

15 tumor necrosis.tw.

16 tumour necrosis.tw.

17 Fc fusion protein*.tw.

18 etanercept*.tw.

19 E?brel*.tw.

20 tnr 001*.tw.

21 tnr001*.tw.

22 adalimumab*.tw.

23 Interleukin 1 Receptor Antagonist Protein/

24 interleukin 1 receptor*.tw.

25 Interleukin1 Receptor*.tw.

26 anakinra*.tw.

27 Kineret*.tw.

28 tocilizumab*.tw.

29 atlizumab*.tw.

30 Actemra*.tw.

31 RoActemra*.tw.

32 r 1569.tw.

33 r1569.tw.

34 rituximab*.tw.

35 Rituxan*.tw.

36 Rituxin*.tw.

37 Reditux*.tw.

38 MabThera*.tw.

39 IDEC-C2B8*.tw.

40 or/6-38

41 5 and 40

42 exp Animals/ not Humans/

43 41 not 42

2) Ichushi

#1 川崎病/TH

#2 川崎病/AL

#3 "kawasaki disease"/AL

#4 "kawasaki syndrome"/AL

#5 "Mucocutaneous Lymph Node Syndrome"/AL

#6 "Mucocutaneous Lymphnode Syndrome"/AL

#7 リンパ節症候群/AL

#8 MCLS/AL

#9 MLNS/AL 4

#10 #1 or #2 or #3 or #4 or #5 or #6 or #7 or #8 or #9

#11 モノクローナル抗体/TH

#12 モノクローナル抗体/AL

#13 "monoclonal antibody"/AL

#14 "monoclonal antibodies"/AL

#15 Infliximab/TH

#16 infliximab/AL

#17 インフリキシマブ/AL

#18 Remicade/AL

#19 レミケード/AL

#20 Inflectra/AL

#21 Revellex/AL

#22 Remsima/AL

#23 Avakine/AL

#24 TNF/AL

#25 "tumor necrosis"/AL

#26 "tumour necrosis"/AL

#27 腫瘍壊死因子/AL

#28 "Fc fusion protein"/AL

#29 Fc融合蛋白質/AL

#30 Fc融合たんぱく質/AL

#31 Fc融合タンパク質/AL

#32 Etanercept/TH

#33 Etanercept/AL

#34 エタネルセプト/AL

#35 Enbrel/AL

#36 Embrel/AL

#37 エンブレル/AL

#38 "tnr 001"/AL

#39 tnr001/AL

#40 Adalimumab/TH

#41 Adalimumab/AL

#42 アダリムマブ/AL

#43 "Interleukin 1 Receptor Antagonist Protein"/TH

#44 "Interleukin 1"/AL and Receptor/AL

#45 "Interleukin-1"/AL and Receptor/AL

#46 "Interleukin 1"/AL and Antagonist/AL

#47 "Interleukin-1"/AL and Antagonist/AL

#48 "インターロイキン1"/AL and (受容体/AL or レセプター/AL)

#49 "インターロイキン-1"/AL and (受容体/AL or レセプター/AL)

#50 "インターロイキン1"/AL and 阻害/AL

#51 "インターロイキン-1"/AL and 阻害/AL

#52 "インターロイキン1"/AL and 拮抗/AL

#53 "インターロイキン-1"/AL and 拮抗/AL

#54 "インターロイキン1"/AL and アンタゴニスト/AL

#55 "インターロイキン-1"/AL and アンタゴニスト/AL

#56 Anakinra/TH

#57 Anakinra/AL

#58 アナキンラ/AL

#59 Kineret/AL

#60 Tocilizumab/TH

#61 tocilizumab/AL

#62 トシリズマブ/AL

#63 atlizumab/AL

#64 アトリズマブ/AL

#65 Actemra/AL

#66 アクテムラ/AL

#67 RoActemra/AL

#68 r-1569/AL

#69 r1569/AL

#70 Rituximab/TH

#71 Rituximab/AL

#72 リツキシマブ/AL

#73 リタキサン/AL

#74 Rituxan/AL

#75 リツキサン/AL

#76 Reditux/AL

#77 MabThera/AL

#78 マブセラ/AL

#79 IDEC-C2B8/AL

#80 #11 or #12 or #13 or #14 or #15 or #16 or #17 or #18 or #19 or #20 or #21 or #22 or #23 or #24 or #25 or #26 or #27 or #28 or #29 or #30 or #31 or #32 or #33 or #34 or #35 or #36 or #37 or #38 or #39 or #40 or #41 or #42 or #43 or #44 or #45 or #46 or #47 or #48 or #49 or #50 or #51 or #52 or #53 or #54 or #55 or #56 or #57 or #58 or #59 or #60 or #61 or #62 or #63 or #64 or #65 or #66 or #67 or #68 or #69 or #70 or #71 or #72 or #73 or #74 or #75 or #76 or #77 or #78 or #79

#81 #10 and #80
